# Comparative efficacy of hydroxychloride and organic sources of zinc, copper, and manganese on egg production and concentration of trace minerals in eggs, plasma, and excreta in female broiler breeders from 42 to 63 weeks of age

**DOI:** 10.1016/j.psj.2024.103522

**Published:** 2024-02-02

**Authors:** Reza Bakhshalinejad, Stephanie Torrey, Elijah G. Kiarie

**Affiliations:** ⁎ Department of Animal Bioscience, University of Guelph, Guelph, Ontario N1G 2W1, Canada; † Trouw Nutrition North America, Puslinch, Ontario, Canada

**Keywords:** broiler breeder, zinc, copper, manganese, reproductive performance, mineral status

## Abstract

Comparative efficacy of hydroxychloride (**HC**) and organic (**OR**) sources of Zn, Cu and Mn on performance of broiler breeders (**BB**) between 42 and 63 weeks of age (**WOA**) was investigated. A total of 408 ♀ Ross 708 and 48 ♂ Yield Plus cockerels were placed in pens (17 ♀ and 2 ♂) housed in 2 rooms (12 pens/room) and allocated to one of 2 diets in a completely randomized block design (n=12). The diets had similar nutrient specifications but differed in Zn, Cu, and Mn sources: 1) **HO**, a blend of 80% HC and 20% OR sources, and 2) **OR,** 100% OR sources. Birds were fed and managed according to breeder guidelines. The egg count was recorded daily and categorized as normal or abnormal. Egg yolk color, albumen height, Haugh unit, eggshell thickness, and eggshell breaking strength were assessed every 4 wk. Individual hen body weight (**BW**) was recorded at 5-wk intervals to determine BW uniformity. At 52 and 63 WOA, the eggs and excreta samples were collected. At the end of the trial, 4 hens per pen were bled for plasma concentration of trace minerals and organs (liver, gizzard, spleen, kidney, and thymus) weight. There were no interactions between source and age on any parameters (*P* > 0.05). There were no main effects of source on egg production, eggshell quality, BW, and organs weight (*P* > 0.05). Hens fed HO diets had darker yolk compared to those fed OR diets (*P* = 0.014). The concentration of Zn in the eggs of OR BB was higher (*P* = 0.022) than for HO birds. However, there were no dietary effects on the concentration of trace minerals in the egg, plasma, and excreta (*P* > 0.05). The results indicated that a mixture of HC and O as sources for Zn, Cu, and Mn was as effective as OR sources in supporting egg production, egg quality, and trace mineral utilization in broiler breeders.

## INTRODUCTION

Trace minerals (**TM**) play a crucial role in supporting the productivity of broiler breeders, as they are indispensable for various digestive and other physiological processes (Wang et al., 2019). Additionally, they contribute to maintaining health and influence the quality of eggs and offspring development (Favero et al., 2013). The impact of deficiencies of specific TM such as zinc (**Zn**), copper (**Cu**), and manganese (**Mn**) on breeder performance and embryo development are significant and well-documented (Araújo et al., 2019). Most TM in commercial diets are derived from inorganic compounds such as sulphates, oxides, and carbonates (Bao and Choct, 2009). Organic (**OR**) sources of TM could potentially replace these inorganic (**InO**) sources. Organic minerals possess the advantage over InO sources in being shielded from chemical interactions with other substances in the intestinal environment due to their lack of dissociation from their respective ligand in the acidic stomach pH (Bao et al., 2007). These characteristics can increase their bioavailability compared to InO sources. Research indicates that organically bound TM can increase the concentration of minerals in the blood or tissues and decrease mineral excretion compared to InO mineral forms (Cao et al., 2002; Świątkiewicz et al., 2014). This phenomenon arises from their ability to prevent the formation of insoluble complexes in the gastrointestinal tract (Abdallah et al., 2009; Mézes et al., 2012). As a result, formulating diets with highly bioavailable OR TM could serve as a strategy to reduce the excretion of TM in animal waste. However, they are considerably more expensive, making their adoption in commercial diets difficult.

Hydroxychloride (**HC**) forms of TM represent a novel category of minerals exhibiting distinct advantages due to their crystalline structure (Hawthorne and Sokolova, 2002). Their robust covalent bonds between hydroxy groups and chloride ions result in a crystallized molecular configuration (Nguyen et al., 2020). This unique molecular structure facilitates a gradual release of minerals during the digestive processing, leading to more efficient absorption and consequently reduced excretion (M'Sadeq et al., 2018). Prior research showcased the superior performance of broilers and laying hens fed HC Zn and Cu compared to those provided with InO counterpart sources (M'Sadeq et al., 2018; Olukosi et al., 2018; Olukosi et al., 2019). One key factor contributing to the heightened effectiveness of HC sources is that only a minimal quantity of these minerals is soluble in water, yet they can achieve full solubility in acidic conditions (Pang and Applegate, 2006). Their crystalline structure and covalent bonds reduce their reactivity to feed ingredients such as vitamins. This advantage becomes particularly apparent when HC TM are concentrated in base mixes, supplements, or dietary formulations (Jasek et al., 2019).

Most of the research in HC TM has focused on their use in broiler chickens and layers. Unfortunately, there is a limited amount of available information in the broiler breeder (**BB**) space. Hence, the primary aim of the present study was to evaluate and compare the effects of a blend of HC and OR sources vs. OR amino sources of Zn, Cu, and Mn on the performance of broiler breeders. This study also aimed to assess their influence on egg quality, organ weight and concentration of TM in eggs, plasma, and excreta.

## MATERIALS AND METHODS

The experimental protocol was approved by the Animal Care Committee of the University of Guelph (AUP #4556), and the birds were cared for in accordance with the guidelines set by the Canadian Council on Animal Care (CCAC, 2009; NFACC, 2016).

### Birds and Feed Management

A total of 480-day-old broiler breeder pullets (Ross 708) and 48 Yield Plus cockerels were procured from Aviagen (Aviagen Inc., Huntsville, AL) through the Ontario Broiler Hatching Egg and Chick Commission. An additional 48-day-old Ross 708 Yield Plus males were placed 20 wk after placement of the main flock and reared for spiking purposes when the flock was at 45 wk of age (**WOA**). This was to enhance the mating frequency as the flock aged in alignment with the industry standards. The rearing facility utilized was designed to be light-tight and featured forced air heating along with evaporative cooling. Throughout the rearing phase, the birds were provided feed through a 152 cm trough feeder, and water was made available through 6 nipples drinker line. During the first 3 d, chicks were subjected to 23 h of light at an intensity of 100 lux (at bird level) with a subsequent 1-h dark period. The photoperiod was then reduced to 19 h at 4 d of age (**DOA**), while maintaining the same light intensity. At 5 and 6 DOA, the photoperiod was adjusted to 14 h and 13 h of light, respectively, at an intensity of 60 lux. Throughout the rearing period, spanning from 10 to 21 WOA, the photoperiod was further decreased to 8 h with light intensity of 20 lux. The vaccination program was as the follows: Newcastle-bronchitis vaccine (administered via spray) at 3 WOA, ILT Vectormune FP-LT-AE (administered via wing web) at 8 WOA, and Newcastle-bronchitis vaccinations at 10 WOA (via spray) and 16 WOA (via intramuscular injection). All aspects of rearing were maintained as closely aligned with industry standards as possible at the University of Guelph Arkell Poultry Research Station. The birds were fed a standard starter diet from d 1 to the 4 WOA and subsequently transitioned to a grower and pre-breeder diet from 5 to 19 and 20 to 21 WOA, respectively. The rearing diets were crumbled and were formulated with InO sources of TM (Floradale Feed Mill Limited, Floradale, ON, Canada). During the starter and grower phases, the birds were provided with a daily feed allowance adjusted on a weekly basis in accordance with the Ross 708 Parent Stock guidelines for body weight (**BW**) and age (Aviagen, 2018). This adjustment was carried out by weighing 25% of the birds at 4, 10, 16, and 21 WOA (Supplementary Table 1).

At 22 WOA, 480 pullets and 48 cockerels were transferred to egg production facility and allocated to 24 floor pens (17 ♀ and 2 ♂). The pens were housed in 2 rooms, with12 pens per room. Allocation of birds to pens considered BW and uniformity. The dimensions of the pens were 9.25 m² (2.5 m × 3.7 m), with 40% of the area covered with wood shavings and the remaining 60% fitted with plastic slats positioned 0.45 m above the floor. Each pen was equipped with a hen trough feeder (measuring 5 cm in depth, 13 cm in width, and 152 cm in length, furnished with rooster-exclusion grills) and a rooster round feeder suspended 60 cm above the ground. All birds had unrestricted access to water through 6 nipples in a nipple drinker line. The photoperiod was adjusted to 12 h of light, maintaining an intensity of 40 lux. The hens were fed a common breeder layer diet until 38 WOA. The daily feed allocation was according to breeder guidelines based on age and BW. Summary of the hen-day egg production (**HDEP**, % was calculated from 25 to 38 WOA and presented in the supplementary Figure 1. The antibiotic-free pelleted diets fed from 22 WOA were provided by Shur-Gain (Shur-Gain, ON, Canada) every 4 wk.

### Experimental Diets

Two diets were formulated to meet or exceed nutrients specifications of BB (Aviagen, 2021a, b). The 2 diets differed on the sources of Zn, Cu, and Mn as follows: 1) **HO**: diet, a blend of 80% HC (Selko IntelliBond, Trouw Nutrition, ON, Canada) and 20% OR from proteinate sources (Selko Optimin, Trouw Nutrition, ON, Canada), or 2) **OR**: 100% OR from amino acid complexes (Zinpro Availa, Eden Prairie, MN). The proportion of HC and OR in the HO dietary group was defined based on proprietary experimentations at Trouw nutrition. The diets were manufactured in pellet form at commercial feed mill (Shur-Gain, St. Mary, ON, Canada) and provided in 2 batches (Table 1).

**Table 1:** Composition of the experimental diet.

	Rooster diet	Hen diet	
Ingredient, %		HO^1^	OR^2^
Corn	45.0	52.3	52.3
Soybean meal	12.1	13.3	13.3
Wheat	38.3	23.1	23.2
Shell rock	-	2.50	2.50
Pork meal	1.54	2.14	2.15
Calcium carbonate	1.27	5.12	5.10
Dicalcium phosphate	0.76	0.07	0.07
Soybean meal oil	-	0.28	0.28
Salt	0.31	0.31	0.32
Vitamin and mineral premix^3^	0.25	0.50	0.50
Methionine	0.18	0.14	0.14
Vitamin E	-	0.07	0.07
Choline chloride	0.06	0.06	0.06
Mold inhibitor	0.15	0.05	0.03
Multi- carbohydrase enzyme	-	0.01	0.01
Phytase	-	0.01	0.01
Lysine	0.04	-	-
Threonine	0.03	-	-

1 Zn, Cu, and Mn supplemented in form of 80% hydroxychloride (IntelliBond) and 20% organic (Optimin).

2 Organic (Availa) sources of Zn, Cu, and Mn

3 Mineral and vitamin premix supplied the following per kg of diet: vitamin A, 13,163 UI; vitamin D3, 4,000 UI; vitamin E, 4,000 UI; vitamin K3, 3.399 mg; vitamin B1, 3.98 mg; vitamin B2, 13.16 mg; pantothenic acid, 21.94 mg; vitamin B6, 7.93 mg; vitamin, B12 0.0395 mg; niacin, 110.04 mg; folic acid, 2.2 mg; Biotin, 0.25 mg; Se, 0.3 mg; Fe, 80 mg; I, 0.81 mg. Cu, Zn and Mn were supplied separately.

### Experimental Procedures and Samples Collection

At 38 WOA, individual BW was recorded, and to achieve a similar BW uniformity between the pen replicates, birds were redistributed to the 24 pens across the 2 rooms. The spiking males were systematically introduced to replace older males when the flock reached 45 WOA. Dietary management followed the guidelines outlined in the breeder guide by Aviagen (2018) from wk 42 to 63. Following a 4-wk adjustment period (38-41 WOA); at the beginning of 42 WOA, the 2 diets were allocated to pens in randomized complete block design to give 12 replicate pens per diet. Individual BW before feeding were recorded at 5-wk intervals for calculation of BW coefficient of variation (**CV**). Egg counts on pen basis was recorded daily at 0800 hrs; a visual inspection was used to differentiate between normal and abnormal eggs. The abnormal eggs included shell-less, broken, misshapen, and dirty eggs. This assessment was conducted daily throughout the experiment. Additionally, 6 settable eggs per pen were selected randomly and weighed each week using a digital balance (Entris 2201‐1S, Sartorius, Göttingen, Germany) accurate to 0.01 gram. Any occurrences of mortality were documented, and the BW was recorded. Feed allotment calculations incorporated the change in the pen population due to mortalities. To evaluate egg quality, 6 eggs were randomly chosen on the select day from each pen at 4-wk intervals. Each egg was individually cracked, and the egg yolk and eggshell weights recorded. The albumen height was measured using a Digital Haugh Tester (OKRA Food Technology LTD, West Bountiful, UT). Also, the eggshell thickness was determined by measuring 3 distinct points of the egg (top, middle, and bottom) using a digital Egg Shell Thickness Gauge (OKRA Food Technology LTD). The average of these 3 measurements was then calculated to determine the overall eggshell thickness. For the determination of eggshell breaking strength, an Egg Force Reader (OKRA Food Technology LTD) equipped with a maximum 50-N load cell and operating at a speed of 1 mm/min was used. In terms of assessing the intensity of egg yolk color, visual examination was carried out using a DSM yolk color fan scale, which consisted of 15 blades with number 1 to 15 (higher values denote more intense color). All analyses pertaining to egg quality were conducted within 12 h following the collection of the eggs.

At 52 and 63 WOA, eggs, plasma and excreta samples were collected for determination of TM concentration. For eggs, 6 fresh eggs were selected randomly from each pen. For plasma, 4 hens were randomly selected per pen and bled via brachial wing vein using a 25-gauge, 1-inch needle and10 mL syringe and immediately transferred into 6 mL BD Vacutainer® Plus Plastic K2EDTA tubes (catalog number: 368381; Becton Dickinson Co, Franklin Lakes, NJ). These samples were immediately placed in an ice box and transported to the laboratory once all the pens had been sampled. Upon arrival, they were promptly subjected to centrifugation (Beckman Coulter Centrifuge, rotor JS-4.2) at 3,000 × *g* for 15 min at 4°C to recover plasma that was stored at -20°C until required for analyses. The method for excreta collection involved employing 2 g/kg of Fe_2_O_3_ (synthetic red iron oxide, 2568, DSM, Ayr, ON, Canada) as an indigestible marker in the hens' diets during the collection period. The excreta samples were gathered twice daily (at 8 am and 6 pm) over a period of 3 consecutive days. The collected samples were then carefully stored in appropriately labeled plastic bags until analysis. At the end of 62 WOA, following 12-h fasting, 4 birds were randomly selected from each pen, individually weighed, and killed by cervical dislocation. The liver, gizzard, kidneys, spleen, and thymus were dissected and weighed by digital balance accurate to 0.01 grams.

### Samples Processing and Laboratory Analyses

Excreta samples were dried in an oven set at 60°C for 48 h (Leung et al., 2018). Fresh eggs were cracked, yolk and albumen contents mixed carefully and an aliquot of 5 g of egg samples were placed in aluminum dishes and placed in oven at 105°C for 24 h (Akbari Moghaddam Kakhki et al., 2020). The samples were then placed in a desiccator and subsequently weighed. The excreta and egg samples were ground in a coffee grinder (CBG5 Smart Grind, Applica Consumer Products Inc., Shelton, CT) and thoroughly mixed. The diet samples were submitted to a commercial laboratory (SGS Canada Inc., Guelph, ON, Canada) for analyses of dry matter, crude protein, and minerals (calcium, phosphorous, potassium, magnesium, sodium, iron, Zn, Cu, and Mn) using Association of Official Agricultural Chemists methods (930.15, 935.11, 935.12, respectively) (AOAC, 2005). An aliquot of 0.5 g of ground egg content and excreta samples were treated with a mixture of 4 mL of trace metal grade nitric acid (Fisher Scientific) and 0.25 mL of trace metal grade hydrochloric acid (Fisher Scientific, Hampton, VA). This mixture was heated to 110°C for 12 h. then stored at 4°C for subsequent analyses. The digested samples of eggs and excreta samples, along with plasma were submitted to the Animal Health Laboratory (**AHL**) at the University of Guelph, Canada for Zn, Cu, Mn, iron, and selenium analyses using inductively coupled plasma mass spectrometry (Agilent 7,900 ICP-MS, Agilent).

### Calculations and Statistical Analyses

The HDEP was calculated on weekly basis using the following formulas: HDEP, % = 100 × (total number of eggs produced in a week / total number of hens presented in the flock on that week). For the computation of egg mass, the egg weight (g) was multiplied by HDEP. The FCR was calculated as the ratio of feed intake to egg mass. The percentage of abnormal eggs was calculated as the number of shell-less, broken, misshapen, and dirty eggs divided the total of eggs produced weekly. The albumen weight was determined by subtracting the sum of the egg yolk and eggshell weights from the total egg weight. The Haugh unit was calculated using the formula: Haugh unit = 100 × log10 (albumen height [mm] − [1.7 × egg weight (g)^0.37^] + 7.57) (Haugh, 1937). The BW uniformity was defined as CV and was calculated by dividing the standard deviation of BW by the average BW of the pen. For statistical purposes, organ weights were represented as a percentage of BW (% BW).

The pen was the experimental unit. The data were assessed for normal distribution using the NORMAL option within the UNIVARIATE and homogeneity of variances was examined using the HOVTEST option in the GLM procedure of SAS. For all parameters except of organ weights, statistical analysis was subjected to a 2-way ANOVA in a 2 dietary sources (HO and OR) × 3 parent age (46, 54, and 62 WOA) factorial arrangement using PROC GLIMMIX procedures of SAS (SAS v. 9.4, SAS Institute Inc., Cary, NC). The model had diet, broiler breeder age and associated interactions as fixed factors and room (block) as variable factor. Tukey procedure was used to separate Least Square Means of for main effect broiler breeder age and interactions. The model of organ weights had diet as fixed factor. Significance was declared at *P* < 0.05.

## RESULTS

The analyzed nutrient composition was comparable with formulated targets (Table 2). The analyzed concentrations of Zn, Cu, and Mn were consistent in both batches (Table 2). The BW and standard deviation of pullets and cockerels during rearing (4-21 WOA) are illustrated in Supplementary Table 1. The HDEP from 25 to 38 WOA (prior to the experiment) and 38 to 41 WOA (during the adaptation phase) is documented in Supplementary Figure 1.

**Table 2:** Calculated and analysed composition of the experimental diets.

Calculated	Rooster diet	Hen diet			
		HO^2^		OR^3^	
AME, kcal/kg	2,725	2,730		2,730	
Dry matter, %	87.3	87.8		87.8	
Crude protein, %	15.3	14.2		14.2	
Crude fat, %	2.77	2.94		2.94	
Crude fiber, %	3.64	2.64		2.65	
Calcium, %	0.88	3.19		3.12	
Total phosphorus, %	0.75	0.44		0.44	
Sodium, %	0.18	0.16		0.16	
Magnesium, %	0.21	0.21		0.21	
Sulfur, %	0.19	0.18		0.18	
Iron, mg/kg	30.0	80.0		80.0	
Iodine, mg/kg	1.25	0.81		0.81	
Selenium, mg/kg	0.30	0.30		0.30	
Zinc, mg/kg	110	120		120	
Copper, mg/kg	30.0	12.0		12.0	
Manganese, mg/kg	120	120		120	


	Rooster diet	Breeder 1		Breeder 2	
Analyzed^1^		HO^2^	OR^3^	HO^2^	OR^3^
Dry matter, %	86.4	88.70	88.54	89.1	88.6
Crude protein, %	16.9	14.5	14.2	14.2	14.6
Calcium, %	1.08	2.43	2.97	2.85	2.50
Total phosphorus, %	0.69	0.41	0.39	0.44	0.45
Potassium, %	0.76	0.52	0.53	0.55	0.58
Sodium, %	0.21	0.15	0.14	0.17	0.17
Magnesium, %	0.23	0.20	0.19	0.19	0.20
Iron, mg/kg	232	328	331	353	376
Zinc, mg/kg	169	147	148	169	169
Copper, mg/kg	40.8	17.0	17.5	27.3	25.6
Manganese, mg/kg	170	143	142	148	143

1 n = 3.

2 Zn, Cu, and Mn supplemented in form of 80% hydroxychloride (IntelliBond) and 20% organic (Optimin).

3 Organic (Availa) sources of Zn, Cu, and Mn.

There were no (*P* > 0.05) were interactions between BB age and source nor the main effects of age and source on the proportion of abnormal eggs, mortality, and BW CV (Table 3). There were no (*P* > 0.05) no interactions between source and BB age or main effect of source on HDEP, egg mass, egg weight, feed intake to egg mass ratio, proportion of abnormal eggs, mortality, hen BW, or BW CV. However, an age effect (*P* < 0.001) was observed on some of these parameters. In this context, HDEP and feed intake to egg mass ratio decreased whereas the egg and body weight increased between 42 and 63 WOA. There were no (*P* > 0.05) no interactions between BB age and source nor main effects of age and source on albumen height, Haugh unit, or eggshell thickness and breaking strength (Table 4). Although there was no interaction between age and source on yolk color, there was a source effect (*P* = 0.014) with birds fed HO having darker yolk colors than OR birds (Table 4). As the bird aged, there was an observed increase in egg yolk color, accompanied with a decrease in eggshell characteristics (*P* < 0.05).

**Table 3:** The effects of feeding hydroxychloride and organic sources of zinc, copper, manganese on egg production and body weight in broiler breeders from 42 to 63 wk of age.

Age, week	Source		SEM^4^			*P*-Value		
	HO^2^	OR^3^	Source	Age	Source × Age	Source	Age	Source × Age
Hen-day egg production (%)								
42–52	53.99^a^	50.67^b^	1.445	1.588	1.955	0.138	0.001	0.673
53–63	43.79^a^	40.33^b^						
42–63	52.36^a^	50.19^b^						
Egg mass production (g/d/hen)								
42–52	36.84^a^	34.34^b^	0.973	1.071	1.324	0.117	0.001	0.590
53–63	30.37^a^	27.64^b^						
42–63	36.03^a^	34.08^b^						
Egg weight (g)								
42–52	68.37^a^	67.71^b^	0.293	0.359	0.507	0.639	0.019	0.699
53–63	69.42^a^	68.60^b^						
42–63	68.81^a^	67.93^b^						
Feed intake to egg mass (g:g)								
42–52	4.45^b^	4.74^a^	0.146	0.162	0.203	0.151	0.001	0.891
53–63	5.35^b^	5.47^a^						
42–63	4.48^b^	4.68^a^						
Abnormal eggs^1^ (%)								
42–52	1.47	1.76	0.247	0.301	0.425	0.468	0.649	0.209
53–63	1.70	1.95						
42–63	1.50	1.87						
Mortality (%)								
42–52	0.18	0.27	0.058	0.069	0.094	0.757	0.970	0.676
53–63	0.17	0.22						
42–63	0.17	0.25						
Body weight (kg)								
42	3.62^a^	3.61^b^	0.024	0.031	0.036	0.102	<0.0001	0.736
47	3.80^a^	3.72^b^						
52	3.90^a^	3.86^b^						
57	3.91^a^	3.87^b^						
62	3.93^a^	3.88^b^						
Body weight CV								
42	10.34	10.41	0.379	0.574	0.787	0.692	0.572	0.976
47	10.30	10.42						
52	10.94	10.82						
57	10.73	10.78						
62	11.10	11.96						

Each value represents the mean of 12 replicates with 17 breeder hens per replicate.

^a-b^ Values within a same row with different superscripts differ significantly at *P* < 0.05.

1 Such as shell-less, broken, misshapen, and dirty eggs.

2 Zn, Cu, and Mn supplemented in form of 80% hydroxychloride (IntelliBond) and 20% organic (Optimin).

3 Organic (Availa) sources of Zn, Cu, and Mn.

4 SEM: standard error of means.

**Table 4:** The effects of feeding hydroxychloride and organic sources of zinc, copper, and manganese on egg quality of broiler breeder in different ages.

Age, week	Source		SEM^3^			*P*-value		
	HO^1^	OR^2^	Source	Age	Source × Age	Source	Age	Source × Age
Albumen Height (mm)								
42–46	8.04	8.02	0.069	0.103	0.140	0.630	0.785	0.994
46–50	8.10	8.03						
50–54	8.21	8.19						
54–58	8.29	8.24						
58–62	8.24	8.20						
42–62	8.20	8.16						
Haugh Unit								
42–46	99.27	99.24	0.293	0.449	0.612	0.575	0.942	0.988
46–50	99.41	99.26						
50–54	99.60	99.59						
54–58	99.81	99.52						
58–62	99.73	99.61						
42–62	99.66	99.40						
Egg Yolk Color								
42–46	7.33^a^	7.25^b^	0.057	0.090	0.124	0.014	<0.0001	0.404
46–50	7.50^a^	7.30^b^						
50–54	7.97^a^	7.80^b^						
54–58	7.78^a^	7.72^b^						
58–62	7.92^a^	7.71^b^						
42–62	7.70^a^	7.67^b^						
Eggshell Thickness (mm)								
42–46	0.441^a^	0.434^b^	0.003	0.005	0.006	0.126	<0.0001	0.889
46–50	0.440^a^	0.435^b^						
50–54	0.434^a^	0.429^b^						
54–58	0.416^a^	0.414^b^						
58–62	0.404^a^	0.390^b^						
42–62	0.425^a^	0.420^b^						
Eggshell Breaking Strength (kgf)								
42–46	3.96^a^	3.93^b^	0.070	0.100	0.134	0.716	0.042	0.847
46–50	3.96^a^	3.92^b^						
50–54	3.93^a^	3.90^b^						
54–58	3.93^a^	3.82^b^						
58–62	3.81^a^	3.80^b^						
42–62	3.85^a^	3.82^b^						

Egg quality data are means of 12 replicates and 6 egg samples per each.

^a-b^ Values within a same row with different superscripts differ significantly at *P* < 0.05.

1 Zn, Cu, and Mn supplemented in form of 80% hydroxychloride (IntelliBond) and 20% organic (Optimin).

2 Organic (Availa) sources of Zn, Cu, and Mn.

3 SEM: standard error of means.

There was no source effect (*P* > 0.05) on liver, gizzard, kidney and lymphoid (spleen and thymus) weight (Table 5). There was no interaction between age and source on the concentration of Zn, Cu, Mn, iron or selenium in the eggs, plasma, and excreta (Table 6). A source effect (*P* = 0.022) was observed for the concentration of Zn in eggs such that birds fed OR had a greater concentration of Zn in the eggs than HO birds (Table 6). The egg concentration of Cu (*P* = 0.022), Mn (*P* = 0.037), and selenium (*P* = 0.026) declined with bird age. There was no source or age effects (*P*>0.05) concentration of TM in the plasma and excreta (Table 6).

**Table 5:** The effects of feeding hydroxychloride and organic sources of zinc, copper, and manganese on organ weights (% BW) of broiler breeder at 63 wk of age.

Item	Source		SEM^3^	*P*-value
	HO^1^	OR^2^		
Liver	1.528	1.597	0.043	0.432
Gizzard	1.109	1.110	0.023	0.875
Kidney	0.387	0.399	0.007	0.742
**Lymphoid organs**				
Spleen	0.065	0.064	0.001	0.862
Thymus	0.033	0.037	0.001	0.514

Each value represents the means of 12 pen replicates with 4 organ samples per each.

All differences among means were no significant.

1 Zn, Cu, and Mn supplemented in form of 80% hydroxychloride (IntelliBond) and 20% organic (Optimin).

2 Organic (Availa) sources of Zn, Cu, and Mn.

3 SEM: standard error of means.

**Table 6:** The effects of feeding hydroxychloride and organic sources of zinc, copper, and manganese on the concentration of trace minerals in eggs, plasma, and excreta of broiler breeder in different ages.

Age, week	Source		SEM^5^			*P*-value		
	HO^3^	OR^4^	Source	Age	Source × Age	Source	Age	Source × Age
**Egg (µg/g of egg)**								
Zinc^1^								
52	69.25^b^	79.08^a^	2.649	3.747	5.298	0.022	0.302	0.808
63	66.25^b^	74.25^a^						
Copper^2^								
52	4.49^a^	4.89^a^	0.104	0.185	0.261	0.062	0.022	0.805
63	4.10^b^	4.41^b^						
Manganese^2^								
52	1.71^a^	1.83^a^	0.064	0.091	0.129	0.820	0.037	0.260
63	1.62^b^	1.53^b^						
Iron								
52	73.17	79.42	2.875	4.067	5.751	0.218	0.058	0.775
63	66.42	70.33						
Selenium^2^								
52	2.83^a^	3.08^a^	0.084	0.119	0.169	0.115	0.026	0.628
63	2.62^b^	2.75^b^						
**Plasma (µg/mL of plasma)**								
Zinc								
52	2.60	2.67	0.270	0.382	0.541	0.721	0.160	0.596
63	3.36	3.02						
Copper								
52	0.19	0.21	0.013	0.018	0.026	0.649	0.927	0.681
63	0.20	0.20						
Manganese								
52	0.03	0.04	0.007	0.010	0.014	0.778	0.168	0.967
63	0.05	0.06						
Iron								
52	5.59	5.82	0.567	0.802	1.134	0.983	0.757	0.796
63	6.05	5.86						
Selenium								
52	0.28	0.29	0.017	0.025	0.035	0.502	0.840	0.814
63	0.26	0.29						
**Excreta (µg/mg of excreta)**								
Zinc								
52	865.83	858.33	90.565	65.720	101.79	0.197	0.831	0.161
63	785.83	966.67						
Copper								
52	113.33	104.08	13.12	8.26	14.36	0.251	0.231	0.286
63	104.50	133.08						
Manganese								
52	855.83	922.50	105.91	67.58	116.20	0.224	0.587	0.802
63	801.67	902.51						
Iron								
52	7,516.67	7,491.67	713.58	737.67	883.90	0.240	0.591	0.227
63	7,008.33	8,800.00						
Selenium								
52	1.05	0.99	0.106	0.117	0.101	0.891	0.245	0.389
63	1.07	1.142						

Each value represents the means of 12 pen replicates with 6 egg/plasma samples per each.

1 ^a-b^ Values within a same row with different superscripts differ significantly at *P* < 0.05.

2 ^a-b^ Values within a same column with different superscripts differ significantly at *P* < 0.05.

3 Zn, Cu, and Mn supplemented in form of 80% hydroxychloride (IntelliBond) and 20% organic (Optimin).

4 Organic (Availa) sources of Zn, Cu, and Mn.

5 SEM: standard error of means.

## DISCUSSION

The main goal of the study was to assess and compare how a blend of HC and OR sources vs. OR amino acid sources of Zn, Cu, and Mn affect the performance of BB from 42 to 63 WOA. Additionally, the study investigated how the source of TM influenced egg quality, organ weights, and TM concentrations in eggs, plasma, and excreta. The results indicated no difference in the production performance in terms of HDEP and egg mass production, aligning with the findings of Xiao et al. (2015) and Jiang et al. (2021). As expected, HDEP decreased as the hen aged; the observed decline of nearly 10% in HDEP over the 10-wk period in our study aligns with the egg production trends observed in a study by Pedro et al. (2021). In that study, there were no differences in HDEP based on TM source in BB fed InO or OR sources of TM but they noted an age dependent decrease in HDEP from 62.1% at 55 WOA to 52.2% at 65 WOA, for InO and OR sources, respectively. The current study indicates that TM source does not influence the number of eggs laid, when the sources differ after peak lay. Regardless of dietary treatment, eggs from older hens (53–63 WOA) were one gram heavier than eggs from younger hens (42–52 WOA). These results agree with the findings of Torres and Korver (2018), who also reported an increase in egg weight as hens aged.

The average BW of hens and roosters before the experiment was heavier than the Aviagen Ross 708 recommendation (Aviagen, 2021b), although the standard deviation was under 14% at 4, 10, 16, and 21 WOA (Supplementary Table 1) in line with industry standards (Aviagen, 2021b). As expected, BW increased as the hens aged, regardless of TM source, and was similar to the target BW (Aviagen, 2021b). Achieving the desired BB target BW for age is a primary benchmark for evaluating the quality of pullets (Thanabalan and Kiarie, 2021; Maina et al., 2024). Producers aim to ensure that 80% of the pullets fall within the designated weight ranges. It is worth noting that flocks with weights significantly below or above the target will reach peak egg production later or earlier, respectively, as indicated by Yuan et al. (1994). Dietary treatment did not influence breeder BW, as expected, because breeder hens were managed according to the guidelines.

The TM source had no effect on egg quality parameters except for the yolk color index. Previous studies for example (Stefanello et al., 2014; Yenice et al., 2015; Xiao et al.,2015; Olukosi et al., 2019) indicated that supplementation of different TM sources had no effect on egg quality. Olukosi et al. (2019) and Yaqoob et al. (2020) reported that the supplementation of TM had a significant effect on egg yolk color in hens fed HC or OR vs. InO sources of Zn, Cu, and Mn. The yolk color index in laying hens is primarily influenced by the carotenoid pigments in the diet. Carotenoids are known to enhance the antioxidant properties of the egg yolk, which may protect the developing embryo from oxidative damage during incubation (Surai, 2002). This protection can translate to higher eggs hatchability rates. Rosa et al. (2012) found that the supplementation of BB diets with canthaxanthin improved the hatchability and fertility rate, and reduced embryo mortality. On the other hand, TM such as Zn and Cu play a critical role in carotenoid absorption and metabolism (Surai, 2002). Opalinski et al. (2012) also documented a significant effect of TM source on yolk color index.

Limited research has explored the impact of TM on organ weights in broiler breeders, and this information could enhance our understanding of overall nutrients utilization. This is particularly relevant when considering the role of TM in energy and protein metabolism as discussed by Olukosi et al. (2019). We did not observe effects of TM sources on liver, gizzard, and kidney weight aligning with findings reported by Nguyen et al. (2020) and van Kuijk et al. (2021) in their investigations of HO and InO sources of Cu and Zn in broiler chickens. Given the known role of TM in immune function, we hypothesized that there might be an effect of source on the thymus and spleen. However, our study revealed that spleen and thymus weights were unaffected by TM sources, suggesting that there may be no discernible difference between the 2 sources of TM in terms of their impact on the immune system.

The TM content in the eggs can vary based on the dietary ingredients, and the overall deposition in different egg components can be influenced by the chemical form of the TM provided in the hen diet (Dobrzañski et al., 2008). Moreover, the concentration of TM in the egg can serve as an indicator of TM bioavailability (Kim et al., 2016). In current study the birds fed OR had a greater concentration of Zn in the eggs than HO birds, which may indicate higher Zn absorption through OR supplementation. However, we did not analyze TM concentration in the different egg components (yolk, albumen, and eggshell), which may have been more relevant. For example, Mabe et al. (2003) highlighted that Mn tends to be primarily distributed within the eggshell, whereas Zn and Cu are more prevalent in the egg yolk. Sun et al. (2012) also observed an increase in Cu concentration in egg albumen and a decrease in yolk when 39-WOA-broiler breeders were fed a combination of organic Cu, Zn, and Mn as opposed to InO sources. The transfer of TM from the hen diet to the egg involves 2 potential pathways: one from the ovary to the yolk, and the other from the oviduct to the albumen, shell membrane, and eggshell, as outlined by Richards (1997). Mateos et al. (2005) suggested that OR minerals have the potential to improve the absorption of other minerals. However, we did not observe enhancements in the retention of other trace mineral retention in the eggs in the current study. These results were consistent with other studies, where dietary OR TM did not affect Zn, Cu, Mn, and iron concentrations in blood (Dobrzañski et al., 2008). While the current study did not show an impact of TM sources on TM excretion, Bao and Choct (2009) demonstrated that incorporating OR TM sources in the diet resulted in 75%, 50%, and 14% reduction in Cu, Zn, and iron excretion, respectively when they compared with InO. Another study reported that a reduction in mineral levels in the excreta was observed when comparing the OR forms vs. InO TM in 66-WOA-laying hens (Yenice et al., 2015).

The primary goal in broiler breeder nutrition is achieving optimal reproductive performance and chick quality. Extended, longitudinal studies on broiler breeders are relatively rare, and the existing literature (Favero et al., 2013; Araújo et al., 2019; Yaqoob et al., 2020) presents varying opinions regarding the effectiveness of utilizing OR or HO TM in commercial broiler breeder diets. The current study found little to no difference between HO and OR sources of TM on broiler breeder egg production and other physiological attributes. Suggesting that a combination of HC and OR was as effective in broiler breeder nutrition as OR based on egg production rate, and eggshell quality, in older breeders.

## Appendix Supplementary materials

Image, application 1
