# Supplementary material for: Comparative efficacy of hydroxychloride and organic sources of zinc, copper, and manganese on egg production and concentration of trace minerals in eggs, plasma, and excreta in female broiler breeders from 42 to 63 weeks of age
Source: Poult Sci. 2024 Feb 2;103(4):103522. doi: 10.1016/j.psj.2024.103522 (PMC10875615; doi:10.1016/j.psj.2024.103522)
Supplement: Supplementary file 1 [file mmc1.docx]

**Comparative efficacy of hydroxychloride and organic sources of zinc, copper, and manganese on egg production and concentration of trace minerals in eggs, plasma, and excreta in female broiler breeders from 42 to 63 weeks of age**

Reza Bakhshalinejad^*^, Stephanie Torrey^*†^, and Elijah G. Kiarie^[[1]](#footnote-1)*^

| **Supplementary** **Table 1.** Pre-experiment body weight and standard deviation accompanied by Ross 708 breeder objective (Aviagen, 2021b). | | | |
| --- | --- | --- | --- |
| Bird Age, Week | BW, g | SD | Objective, g |
| **Pullets** |  |  |  |
| 4 | 412.1 | 13.9 | 400 |
| 10 | 1074.7 | 12.1 | 940 |
| 16 | 1636.1 | 11.2 | 1480 |
| 21 | 2231.2 | 10.8 | 2100 |
| **Cockerels** |  |  |  |
| 4 | 856.9 | 13.3 | 755 |
| 10 | 1713.4 | 12.7 | 1670 |
| 16 | 2663.5 | 11.0 | 2420 |
| 21 | 3427.7 | 10.7 | 3195 |
|  | | | |

**Supplementary Figure 1:** Hen-Day egg production from 25 to 38 WOA (pre-experiment) and 38 to 41 WOA (adaptation phase).

| **Supplementary** **Table 2.** Composition of the diet before the main experiment. | | | | | |
| --- | --- | --- | --- | --- | --- |
|  | Starter  (0-4 WOA) | Grower  (5-19 WOA) | Pre-breeder  (20-21 WOA) | Pre-Experiment  (22-38 WOA) | Rooster  (20-38 WOA) |
| **Ingredient, %** |  |  |  |  |  |
| Corn | 45.89 | 49.56 | 44.30 | 52.29 | 45.00 |
| Wheat | 18.50 | 31.00 | 21.00 | 23.20 | 38.31 |
| Soybean meal | 10.50 | 2.00 | 8.70 | 13.28 | 12.10 |
| Bakery meal | 10.00 | - | 9.40 | - | - |
| Pork meal | 7.10 | 5.80 | 8.00 | 2.15 | 1.54 |
| Poultry meal | 0.12 | 0.12 | 0.12 | - | - |
| Canola | 5.00 | 5.00 | - | - | - |
| Shell rock | - | - | - | 2.50 | - |
| Barley | - | 4.00 | - | - | - |
| Limestone | 1.10 | 1.35 | 7.15 | - | - |
| Salt | 0.07 | 0.20 | 0.07 | 0.32 | 0.31 |
| Tallow | - | - | 0.50 | - | - |
| Soybean meal oil | - | - | - | 0.28 | - |
| Calcium carbonate | - | - | - | 5.10 | 1.27 |
| Dicalcium phosphate | - | - | - | 0.07 | 0.76 |
| Monocalcium phosphate | 0.70 | 0.45 | 0.40 | - | - |
| L-Lysine | 0.40 | 0.15 | - | - | 0.04 |
| Methionine | 0.31 | 0.19 | 0.20 | 0.14 | 0.18 |
| Threonine | 0.07 | - | - | - | 0.03 |
| Vitamin E | 0.10 | - | - | 0.07 | - |
| Sodium sesquicarbonate | 0.09 | 0.12 | 0.08 | 0.03 | 0.15 |
| Choline chloride | 0.05 | 0.06 | 0.08 | 0.06 | 0.06 |
| Multi-Carbohydrase enzyme | - | - | - | 0.01 | - |
| Phytase | - | - | - | 0.01 | - |
| Vitamin and mineral premix^1^ | - | - | - | 0.50 | 0.25 |
| Total | 100 | 100 | 100 | 100 | 100 |
| **Calculated nutrient content** |  |  |  |  |  |
| Metabolizable energy, kcal/kg | 2,800 | 2,800 | 2,800 | 2,800 | 2,800 |
| Dry matter, % | 86.32 | 86.41 | 87.03 | 87.81 | 87.34 |
| Crude protein, % | 18.60 | 15.10 | 16.00 | 14.16 | 15.26 |
| Crude fat, % | 3.60 | 3.10 | 3.70 | 2.94 | 2.77 |
| Crude fiber, % | 3.70 | 4.30 | 5.00 | 2.65 | 3.64 |
| Calcium, % | 1.05 | 1.00 | 3.21 | 3.12 | 0.88 |
| Phosphorus, % | 0.75 | 0.72 | 0.67 | 0.44 | 0.75 |
| Sodium, % | 0.17 | 0.17 | 0.17 | 0.16 | 0.18 |
| ^1^Mineral and vitamin premix supplied the following per kg of diet: vitamin A, 13,163 UI; vitamin D3, 4,000 UI; vitamin E, 4,000 UI; vitamin K3, 3.399 mg; vitamin B1, 3.98 mg; vitamin B2, 13.16 mg; pantothenic acid, 21.94 mg; vitamin B6, 7.93 mg; vitamin, B12 0.0395 mg; niacin, 110.04 mg; folic acid, 2.2 mg; Biotin, 0.25 mg; Cu, 120 mg; Mn, 120 mg; Zn, 12 mg; Fe, 80 mg; Se, 0.3 mg; I, 0.81 mg. | | | | | |

1. Correspondences: ekiarie@uoguelph.ca [↑](#footnote-ref-1)
